# Validity testing of the conspiratorial thinking and anti-expert sentiment scales during the COVID-19 pandemic across 24 languages from a large-scale global dataset

**DOI:** 10.1017/S0950268822001443

**Published:** 2022-09-12

**Authors:** Hyemin Han, Angélique M. Blackburn, Alma Jeftić, Thao Phuong Tran, Sabrina Stöckli, Jason Reifler, Sara Vestergren

**Affiliations:** 1 Educational Psychology Program, University of Alabama, Tuscaloosa, AL, USA; 2 Department of Psychology and Communication, Texas A&M International University, Laredo, TX, USA; 3 Peace Research Institute, International Christian University, Tokyo, Japan; 4 Department of Psychology, Colorado State University, Fort Collins, CO, USA; 5 Department of Consumer Behavior, University of Bern, Bern, Switzerland; 6 Department of Politics, University of Exeter, Exeter, UK; 7 School of Psychology, Keele University, Keele, UK

**Keywords:** Anti-expert sentiments, conspiratorial thinking, COVID-19, international survey, validation

## Abstract

In this study, we tested the validity across two scales addressing conspiratorial thinking that may influence behaviours related to public health and the COVID-19 pandemic. Using the COVIDiSTRESSII Global Survey data from 12 261 participants, we validated the 4-item Conspiratorial Thinking Scale and 3-item Anti-Expert Sentiment Scale across 24 languages and dialects that were used by at least 100 participants per language. We employed confirmatory factor analysis, measurement invariance test and measurement alignment for internal consistency testing. To test convergent validity of the two scales, we assessed correlations with trust in seven agents related to government, science and public health. Although scalar invariance was not achieved when measurement invariance test was conducted initially, we found that both scales can be employed in further international studies with measurement alignment. Moreover, both conspiratorial thinking and anti-expert sentiments were significantly and negatively correlated with trust in all agents. Findings from this study provide supporting evidence for the validity of both scales across 24 languages for future large-scale international research.

## Introduction

Before and throughout the COVID-19 pandemic, beliefs in conspiratorial theories and negative attitudes about experts have been on the rise. Conceptually, conspiratorial thinking is an increased likelihood to view the world in conspiratorial terms and attribute the causes of events to groups acting in secret for personal benefit against the common good [1, 2]. Anti-expert sentiments, a phenomenon often studied alongside conspiratorial thinking, is a form of anti-elitist and anti-intellectualism, which is marked by distrust of individuals who claim to be experts or have credentials about a topic [2, 3]. The rise in conspiratorial thinking and anti-expert sentiments in recent times may occur in part due to increases in use of conspiracy theories for political gain [3–5], the rise in confirmation bias in social media circles [6], inconsistencies in public health information [7] or the fact that conspiracy theories proliferate during societal crises and times of uncertainty [8]. Given the potential harm by conspiratorial thinking and anti-expert sentiments, it is critical to have a rapid and effective global tool to assess both types of thinking in order to implement mitigation plans to improve science-driven public health and policy decisions.

### Need for cross-language scale validity for rapid data collection during a global health crisis

Conspiracy theories can influence social and political behaviours [1, 3, 9, 10] and result in undesirable and even catastrophic social outcomes [3, 7, 11]. Of particular interest for an international health crisis such as the COVID-19 pandemic is that believing conspiracy theories was linked to vaccine hesitancy [6], reduced compliance with containment measures [7, 12, 13] and reduced behaviours linked to civic and social responsibility [14]. Specifically, doubters and deniers of COVID-19 risk tended to believe conspiracy theories related to the pandemic, expressed anti-elitist sentiments and reported low compliance with measures to reduce the spread of the virus [12]. Low trust in institutions, including the scientific community, is also linked to vaccine hesitancy as well as compliance with preventive measures in general [15–17]. Finally, conspiracy theories and negative attitudes towards experts have other detrimental effects such as increasing uncertainty and discrimination against marginalised groups [9].

Overall, both conspiracy theories and negativity towards experts can have lasting impacts on the trajectory of a global (health) crisis. Therefore, a consistent method of measuring conspiratorial thinking and anti-expert sentiments across languages is needed, especially when considering political and public health events on a global scale. Reliable means to rapidly assess these beliefs across countries are necessary to implement mitigation strategies [3]. This is particularly critical, as interventions to reduce these beliefs with accompanying behaviours may be fairly straightforward and rapidly implemented [16].

There are an endless number of conspiracy theories that attract individuals across different demographics [e.g. 12, 18], so a singular scale which measures specific conspiracy theory beliefs is difficult to generalise. Uscinski *et al*. [1] developed the Conspiratorial Thinking Scale (CTS) assessing individuals' general disposition towards believing conspiracy theories. Previous work showed that individuals with conspiratorial thinking are also more likely to report anti-expert beliefs, and vice versa [14, 19]. As such, the COVIDiSTRESSII Consortium developed an Anti-Expert Sentiment Scale (AESS) [20] to gauge individuals' levels of distrust in expert consensus.

However, these scales have yet to be validated across different languages. This is critical because a general CTS in different languages provides a way to compare conspiracy theorising across political contexts in a way that studying specific conspiracy theories could not. Likewise, the AESS was designed to be generalisable across countries and contexts. The CTS and AESS are the shortest of the available scales and, once validated across languages, provide scholars with a cost-effective and efficient way of measuring conspiratorial thinking and anti-expert sentiment in multi-country studies.

### Relationship between conspiratorial thinking and anti-expert beliefs, and trust as a mean to validate scales

Robust associations have been reported between general conspiratorial thinking and trust in government, science and public health institutions [2, 13]. Moreover, trust in an institution, whether political or scientific, was tightly coupled with conspiratorial thinking specifically related to that institution [2, 21, 22]. For instance, a strong correlation has been observed between belief in conspiracy theories related to vaccines and reduced trust in science and institutions [6]. Likewise, trust in government mediated the inverse relationship between conspiratorial thinking and compliance with social distancing behaviours to reduce the spread of disease [13]. The relationship between trust and conspiratorial thinking is so robust that mere exposure to a conspiracy claim has been shown to negatively affect trust in government institutions, even of institutions that were not connected to the conspiracy theory [23].

The likelihood of believing a particular conspiracy theory appears to be driven to some degree by exposure to information related to the conspiracy (e.g. within one's social network), while also heavily driven by a combination of general conspiratorial thinking and trust [1], which in turn can affect how one perceives the information they are exposed to. Also, studies that included diverse psychological constructs and demographics documented denialism of expert information as the strongest predictor of believing in COVID-19 conspiracy theories as measured by the CTS and partisan and ideological motivations [14]. Partisanship appears to drive the direction of conspiratorial thinking in such a way that members of one political party are more inclined to believe conspiracy theories about another, and vice versa, even when the degree of general conspiratorial thinking did not differ between political parties [1]. In other words, the degree of trust in an institution is linked to conspiratorial thinking related to that institution, and perhaps to other government institutions and services more broadly [23]. Hence, a negative association of conspiratorial thinking and anti-expert beliefs with trust could be expected.

### This study

In this paper, we tested the validity of scales capturing conspiratorial thinking and anti-expert sentiments that may influence behaviours related to public health during an epidemic or pandemic. In particular, we used two scales: the 4-item CTS adapted from Uscinski *et al*. [1] and a 3-item AESS designed by the COVIDiSTRESSII Consortium [20] and tested their cross-language validity. While a number of conspiracy belief scales have been tested [24–27], we selected the CTS due to its face and content validity. Given that the CTS has been used in various previous studies examining conspiratorial thinking within the context of COVID-19 research, it is possible to assume that its validity has been supported by findings from such studies. However, so far, the scale has been primarily used within the US context, it might need to be tested in diverse settings. The COVIDiSTRESSII Consortium opted to adapt a short new scale that fully captured the concept of anti-expert sentiments using items created by a co-author, and which included three questions about belief in expert knowledge compared to confidence in one's own knowledge.

Assuring the measurement validity of the two scales in different languages is the first step to take before conducting international research on the topic. In addition, during the survey process, participants were presented with survey forms in different languages depending on their first language. Hence, we focused on the measurement validity across different languages in the present study. We tested the measurement invariance and alignment of these scales across 24 languages and dialects using the COVIDiSTRESSII Global Survey dataset. In addition, we also examined whether the measurement model can be applied to individual language groups. If the measurement model is valid within each individual group, then researchers who intend to collect data from a single language group but do not intend to conduct international comparison would be able to use the measures written in their own language.

The measurement invariance test was conducted to examine whether the scales in different languages were designed to measure the same construct in the same measurement structure across different languages [28]. The presence of scalar invariance, which assumes the same factor loadings and intercepts across groups, is essential to assure the quality of cross-national research using the scales [29]. Measurement alignment was performed to address the potential issue of measurement non-invariance reported by the measurement invariance test as done in prior COVID-19-related international survey studies if needed [15]. The measurement alignment process was expected to address non-invariance so that researchers would be able to conduct cross-national comparison. Whether the measures written in a single language can be employed in studies focusing on one language group, not international comparison, was also examined during the invariance test process.

We then assessed the convergent validity of each scale by testing the expected correlations between both CTS and AESS scales and items measuring trust in institutions. We predicted negative correlations between both scales and different trust items. In particular, because trust in political entities is related to conspiratorial thinking [9], we predicted a negative correlation between the CTS and trust in one's national parliament or government. We also predicted negative correlations between the AESS and trust in the scientific community and the World Health Organization (WHO). Positive correlations between CTS and AESS and negative correlations of each scale with trust, as demonstrated in previous literature, would indicate that the scales are measuring the intended constructs.

## Methods

### Dataset

The COVIDiSTRESSII Global Survey is a pre-registered, large-scale international survey dataset collected online by a consortium of over 150 international researchers who used local recruitment methods and snowball sampling to recruit anonymous volunteers from 137 countries across the globe [20]. This survey was administered online from 28 May through 29 August 2021. The data collection process was initially reviewed and approved by the Research, Enterprise and Engagement Ethical Approval Panel at the University of Salford (IRB number: 1632). The cleaned dataset included responses from 15 740 participants from 137 countries (see [20] for further details about the data collection and cleaning processes).

As measurement invariance test and measurement alignment involve confirmatory factor analysis (CFA), following statistical guidelines, we analysed responses in language groups where *n* ≥ 100 [30, 31]. After excluding language groups with *n* < 100, we retained 12 261 responses in 24 language groups for further analysis. Demographics of the participants are presented in Table S1.

### Materials

All items were first prepared in English. Then, the English version was translated and back translated into various languages by researchers with native language skills.

#### Conspiratorial Thinking Scale

At the beginning of the survey section addressing conspiratorial thinking and anti-expert sentiments, participants were presented with the following statement: ‘We will now present a few statements about the COVID-19 virus and about you. Please read the statements and indicate to what extent you agree with them’. Then, conspiracy thinking was measured with four items. These four items were slightly modified from Uscinski *et al*. [1]. The four items were: ‘much of our lives are being controlled by plots hatched in secret places’, ‘even though we live in a democracy, a few people will always run things anyway’, ‘the people who really “run” the country are not known to the voter’, ‘big events like wars, recessions, and the outcomes of elections are controlled by small groups of people who are working in secret against the rest of us’. Responses were anchored to a 7-point Likert scale, ‘1: Strongly disagree to 7: Strongly agree’.

#### Anti-Expert Sentiment Scale

Based on findings relating conspiratorial thinking and anti-expert sentiments [1, 14], the items for the AESS were formulated by experts in the COVIDiSTRESSII Consortium based on previous research, e.g. [1–3]. The items consist of: ‘I am more confident in my opinion than other people's facts’, ‘most of the time I know just as much as experts’, ‘experts really don't know that much’. Answers were anchored to a 7-point Likert scale, ‘1: Strongly disagree to 7: Strongly agree’.

#### Trust

To test convergent validity of the two scales, we also collected data about trust in agents that are addressing the COVID-19 pandemic. Following methods from Lieberoth *et al*. [32], seven items were used to survey trust in these seven agents: parliament/government; police; civil service; health system; the WHO; government's effort to handle Coronavirus; scientific research community. Responses were anchored to an 11-point scale, ‘10%: No trust to 90%: Complete trust’.

### Analysis plan

#### Measurement invariance test

To examine whether the two scales were valid across different languages, we performed a measurement invariance test with *lavaan* [33]. Before examining the cross-language validity of the scales, their internal consistency was tested in terms of Cronbach's *α*. Following the internal consistency testing the theoretical measurement model of each scale was tested with CFA while setting the language as a group. Because responses to the items were anchored to a 6-point Likert scale, we employed the diagonally weighted least squares estimator as suggested by DiStefano *et al.* [34].

Measurement invariance was examined in terms of whether model fit indicators, i.e. RMSEA, SRMR, CFI, changed significantly when different levels of model constraints were applied [31]. We tested four different levels of measurement invariance: configural, metric, scalar and residual invariance [29]. First, the most lenient invariance, configural invariance only assumes the equal measurement structure across different groups. The presence of configural invariance suggests that the examined factor structure can be validly applied across different groups [37, 38]. Thus, if configural invariance is achieved, the examined scale can be used within one specific group with the tested measurement model provided cross-group comparison is not conducted. Second, metric invariance additionally assumes equal loadings. Third, achievement of scalar invariance requires equal intercepts. Fourth, the strictest invariance, residual invariance, assumes the presence of equal residuals. In general, scalar invariance is a minimum requirement for between-group comparison. In the case of metric invariance, we required ΔRMSEA < +0.015, ΔSRMR < +0.030 and ΔCFI > −0.01. For the other invariance levels, we examined whether ΔRMSEA < +0.015, ΔSRMR < +0.015 and ΔCFI > −0.01 [28].

#### Measurement alignment

If at the least scalar invariance was not achieved, we performed measurement alignment to address the existing measurement non-invariance between different languages. Measurement alignment was performed with the *sirt* package [35]. It addresses non-invariance by adjusting factor loadings, intercepts and group means across different groups [29].

After conducting measurement alignment, we examined whether the alignment process was successful with two *R*^2^ indicators, *R*^2^_loadings_ and *R*^2^_intercepts_. Those *R*^2^ values indicate the extent of non-invariance in factor loadings and intercepts, respectively [36]. The value *R*^2^ = 1.00 indicates that 100% of non-invariance was successfully absorbed through alignment while *R*^2^ = 0.00 means that none of non-invariance was resolved. In general, whether less than 25% of non-invariance remains after alignment is regarded as a criterion to determine the success of alignment [36]. Thus, we examined whether both *R*^2^ values were 75% higher in the present study. If both values exceeded the cut-off, we assumed that non-invariance was successfully addressed, and thus, scalar invariance was achieved through alignment.

In addition, we also examined whether there were any significant unique item parameters in both the factor loadings and intercepts across language groups, which were deemed to demonstrate significantly deviated loadings or intercepts relative to other groups. This process was conducted by performing *invariance_alignment_constraint* implemented in *sirt*. The function was developed to adjust factor loadings and intercepts across groups so that the aligned model can absorb non-invariances through measurement alignment. Once more than 25% of item parameters reported significant unique parameters, we deemed that there was significant measurement non-invariance either in loadings or intercepts. The 25% cut-off value was employed by Asparouhov and Muthén [36].

Once measurement alignment was completed, we calculated factor scores with adjusted factor loadings and intercepts for each language group. We used the factor scores for further analyses. Furthermore, we tested whether measurement alignment was capable of producing consistent outcomes. For repetitive cross-validation, we employed a simulation test, which was originally implemented in the format of Monte Carlo simulation for cross-validation of measurement alignment [39]. We generated a simulation dataset with *N* = 100, 200 and 500 per group. Then, we performed measurement alignment with the generated dataset and examined whether it produced outcomes consistent with CFA. The consistency was quantified in terms of Spearman correlation coefficient between factor mean scores estimated by alignment and CFA (see supplementary materials in Lieberoth *et al*. for further methodological details [32]). The same simulation process was performed 500 times with multiprocessing for cross-validation with improved computational power [40]. Following Muthén and Asparouhov, which employed the same procedure, we assumed that a mean correlation value ≥0.95 means good consistency and reliability of alignment [39]. For additional information, correlation between factor variances estimated by measurement alignment and CFA was also examined.

#### Correlation analysis

We examined the correlation between conspiratorial thinking and anti-expert sentiments, and seven trust items to test the convergent validity of the two scales. In the case when measurement alignment was conducted, we employed factor scores that were calculated with adjusted factor loadings and intercepts for the correlation analysis to address the issue of measurement non-alignment [17]. For additional information, we also examined the correlation between factor scores estimated without alignment and trust variables as well.

## Results

### Measurement invariance test

When the internal consistency of each scale was examined in terms of Cronbach's *α*, both the CTS (*α* = 0.85) and AESS (*α* = 0.74) reported at least acceptable consistency. Findings from the measurement invariance test are presented in Table 1.

**Table 1.** Results from the measurement invariance test

	RMSEA	SRMR	CFI	ΔRMSEA	ΔSRMR	ΔCFI
Conspiratorial thinking						
Configural invariance	0.072	0.037	0.993			
Metric invariance	0.083	0.021	0.976	0.011	−0.015	−0.016
Scalar invariance	0.155	0.118	0.868	0.072	0.060	−0.108
Anti-expert sentiment						
Configural invariance	0.000	0.000	1.000			
Metric invariance	0.064	0.040	0.978	0.064	0.040	−0.022
Scalar invariance	0.157	0.101	0.735	0.093	0.061	−0.243

As shown, although configural invariance, which supports the equal measurement structure across languages, was achieved in both scales, metric invariance as well as scalar invariance were not achieved due to changes in RMSEA, SRMR and CFI exceeding the cut-off values. Although the raw values of RMSEA (~0.08), SRMR (<0.08) and CFI (≥0.90) *per se* were seemingly acceptable, the changes exceeded the set thresholds (i.e. ΔRMSEA < +0.015, ΔSRMR < +0.030, ΔCFI > −0.01). Hence, we conducted measurement alignment to address the measurement non-invariance issue.

### Measurement alignment

We performed measurement alignment for the two scales to address non-invariance to enable future cross-national investigations using the scales. First, when measurement alignment was performed for the CTS, the resultant *R*^2^_loadings_ = 0.97 and *R*^2^_intercepts_ = 0.99. Second, in the case of the AESS, *R*^2^_loadings_ = 0.85 and *R*^2^_intercepts_ = 0.99.

Furthermore, our inspection of item parameters also showed that no more than 25% of item parameters reported unique parameters. In the case of the CTS, 6.2% of factor loadings and 19.8% of intercepts reported significant unique item parameters (see Tables S2 and S3 for the groups reported significant item parameters in CTS factor loadings and intercepts, respectively). When the AESS was examined, 6.9% of factor loadings and 19.4% of intercepts demonstrated significant unique item parameters (see Tables S4 and S5 for the groups reported significant item parameters in AESS factor loadings and intercepts, respectively). In all cases, the proportions were below the cut-off value, 25%. These findings support the point that measurement non-invariance in both factor loadings and intercepts were successfully addressed.

The simulation test for consistency check reported that measurement alignment was capable of producing consistent and reliable outcomes across repetitions. In all cases, *N* = 100, 200 and 500, the mean correlation between the factor mean scores estimated by alignment and original CFA exceeded 0.95 (see Cor (mean) in Table 2). As proposed by Muthén and Asparouhov, the good correlation coefficient resulting from the simulation test suggests that measurement alignment was able to produce consistent outcomes, in terms of factor loadings and intercepts, across trials.

**Table 2.** Repetitive simulation test results

	*N* = 100		*N* = 200		*N* = 500	
	*M*	s.d.	*M*	s.d.	*M*	s.d.
CTS						
Cor (mean)	0.96	0.02	0.97	0.01	0.97	0.01
Cor (var)	0.85	0.05	0.85	0.04	0.85	0.03
AESS						
Cor (mean)	0.95	0.01	0.96	0.01	0.96	0.01
Cor (var)	0.62	0.15	0.69	0.12	0.71	0.11

*Note*: Cor (mean): correlation between factor mean scores estimated by measurement alignment and CFA across repetitions. Cor (var): correlation between factor variances estimated by measurement alignment and CFA across repetitions.

For additional information, factor loadings and intercepts per group before and after measurement alignment are reported in the Supplementary materials. Factor loadings and intercepts in each group estimated by multigroup CFA are reported in Tables S6 and S7, respectively. Those resulting from measurement alignments are demonstrated in Tables S8 and S9, respectively.

### Correlation analysis

The result of the correlation analysis is presented in Table 3. In Table 3, CTS and AESS factor scores were estimated with factor loadings and intercepts adjusted through measurement alignment. The same correlation pattern between variables was also found when factor scores estimated without alignment were examined (see Table S10).

**Table 3.** Correlation between conspiratorial thinking and anti-expert sentiment with trust

	1	2	3	4	5	6	7	8
1. Conspiratorial thinking								
2. Anti-expert sentiment	0.45							
3. Trust in parliament/government	−0.44	−0.17						
4. Trust in police	−0.40	−0.18	0.70					
5. Trust in civil service	−0.42	−0.20	0.74	0.76				
6. Trust in health system	−0.39	−0.26	0.57	0.66	0.69			
7. Trust in the WHO	−0.37	−0.31	0.43	0.38	0.48	0.50		
8. Trust in governmental effort	−0.40	−0.17	0.79	0.61	0.67	0.57	0.46	
9. Trust in scientific research community	−0.40	−0.41	0.39	0.39	0.47	0.55	0.61	0.46

*Note*: Conspiratorial thinking and anti-expert sentiment scores were calculated based on results from measurement alignment. In all cases, *p* < 0.001 after applying false discovery rate correction.

## Discussion

When measurement invariance was tested, although both scales achieved configural invariance, they were not able to demonstrate metric invariance. Given scalar invariance is required for multigroup comparison, the two scales might not be used for such comparison without additional processing. The results of measurement alignment suggest that the process was able to handle the measurement non-invariance issue in a satisfactory manner for both the CTS and AESS. The majority of the non-invariance existing in loadings (≥85%) and intercepts (≥99%) across different languages was absorbed by adjusting loadings and intercepts. Also, in all cases, less than 25% of item parameters demonstrated significant unique parameters. Hence, although scalar invariance was not achieved when measurement invariance test was conducted initially, we found that both scales can be employed in further international studies with measurement alignment. Furthermore, the repetitive simulation results suggest that measurement alignment was capable of producing consistent outcomes across trials in the present study.

One point to note is that configural invariance was achieved in both scales, so researchers who intend to collect data from one language group can use the scales if they do not compare scores across different language groups. Given the presence of configural invariance means that the same factor structure is valid across different groups [37, 38], using the scales for further analyses within one group can be justifiable even without alignment. However, given scalar invariance was not achieved, if international comparison involving multiple languages becomes a goal, then measurement alignment may be required.

The result of the correlation analysis also provides additional evidence supporting the validity of the two scales. Both conspiratorial thinking and anti-expert sentiments were significantly and negatively associated with trust in all agents. The finding was consistent with prior research regarding how conspiratorial thinking and objective vaccine knowledge within the context of COVID-19 (e.g. ‘the government is trying to cover up the link between vaccines and autism’) were associated with trust in science and institutions [6]. The pattern of effects was also consistent with previous literature, with the strongest correlations within institutions and significant correlations across all trust agents [23]. That is, the negative correlation between the CTS and trust in one's national parliament or government is consistent with previous literature indicating that trust in political entities is related to conspiratorial thinking [21]. Likewise, negative correlations between the AESS and trust in experts – the scientific community and the WHO – are consistent with previous literature [19]. The similar correlation pattern was found when correlation analysis was performed with factor scored without alignment. This may provide additional evidence supporting that the two scales can be used within one language group even without conducting measurement alignment when international comparison is not performed.

## Conclusions

To summarise, we validated the 4-item CTS and 3-item AESS across 24 languages and dialects using the COVIDiSTRESSII Global Survey dataset (*N* = 12 261). Although scalar invariance was not achieved when the measurement invariance test was conducted initially, we found that both scales can be employed in further international studies with measurement alignment. For future studies focusing on only one language group, not international comparison, researchers may use the two scales composed in one language for their analyses since configural invariance was achieved and the measurement model was validated across groups. Moreover, both conspiratorial thinking and anti-expert sentiments were significantly correlated with each other and negatively correlated with trust in all agents. As both conspiratorial thinking and anti-expert sentiments have negative implications for political events and public health and safety, having a consistent measure across languages is critical for rapid data collection in the face of an international disaster or public health crisis. The findings from this study provide evidence supporting the validity of both scales across 24 languages for future large-scale international research, and can thus be used to measure these factors during a global health crisis such as the COVID-19 pandemic.

## Data Availability

All data files and analysis scripts for this study can be found under this link: https://github.com/hyemin-han/COVIDiSTRESS2_belief_scales.

## Supplementary material

For supplementary material accompanying this paper visit https://doi.org/10.1017/S0950268822001443. click here to view supplementary material
