# Supplementary material for: Validity testing of the conspiratorial thinking and anti-expert sentiment scales during the COVID-19 pandemic across 24 languages from a large-scale global dataset
Source: Epidemiol Infect. 2022 Sep 12;150:e167. doi: 10.1017/S0950268822001443 (PMC9530382; doi:10.1017/S0950268822001443)
Supplement: Supplementary file 1 [file S0950268822001443sup001.pdf]

Supplementary Materials

Supplementary Tables

Table S1

Demographics of participants from language groups where n ≥ 100

		Gender			Age (years)		Education level						
	N	Female	Male	Other/Would rather not say	Mean	SD	Doctorate	University	Some university/ college	≥ 12 years of school	≥ 9 years of school	≥ 6 years of school	None
Total	12,261	67.03%	32.00%	0.97%	37.10	14.56	5.96%	48.53%	25.67%	16.51%	2.26%	0.43%	0.64%
Bulgarian	253	75.10%	24.51%	0.40%	40.80	16.38	5.56%	47.62%	34.13%	12.30%	0.00%	0.00%	0.40%
Czech	304	70.72%	27.63%	1.64%	33.52	11.32	4.93%	48.68%	30.92%	15.13%	0.00%	0.33%	0.00%
German	620	64.84%	34.68%	0.48%	44.29	18.53	6.94%	47.74%	18.06%	23.23%	3.39%	0.16%	0.48%
English	1,246	66.35%	32.45%	1.20%	30.88	11.49	10.03%	52.81%	34.11%	2.65%	0.40%	0.00%	0.00%
Spanish (Colombia)	470	66.60%	32.98%	0.43%	40.06	12.27	6.61%	76.33%	12.79%	2.99%	0.85%	0.21%	0.21%
Spanish (Costa Rica)	191	71.20%	27.23%	1.57%	36.54	10.91	1.05%	83.77%	10.99%	2.62%	1.05%	0.52%	0.00%

Spanish (Ecuador)	218	66.97%	31.65%	1.38%	33.14	11.23	3.67%	72.94%	19.27%	2.75%	0.00%	0.92%	0.46%
Spanish (Spain)	587	67.58%	31.91%	0.51%	40.63	13.54	17.72%	57.92%	21.12%	2.56%	0.68%	0.00%	0.00%
Spanish (Guatemala)	181	84.53%	15.47%	0.00%	36.67	14.89	3.31%	64.09%	29.28%	2.76%	0.00%	0.55%	0.00%
Spanish (Uruguay)	220	86.36%	13.64%	0.00%	42.65	13.00	6.82%	74.09%	11.36%	5.91%	1.36%	0.00%	0.45%
Spanish (Honduras)	314	66.56%	32.17%	1.27%	25.31	8.24	0.64%	18.47%	64.01%	14.97%	0.64%	0.64%	0.64%
Estonian	219	86.70%	13.30%	0.00%	39.32	10.50	1.38%	56.42%	22.02%	19.27%	0.92%	0.00%	0.00%
Finnish	847	79.57%	19.13%	1.30%	46.32	14.38	4.15%	54.92%	19.10%	16.96%	2.61%	1.66%	0.59%
Italian	279	73.02%	26.62%	0.36%	45.23	16.07	7.27%	47.64%	22.55%	20.36%	1.82%	0.36%	0.00%
Japanese	2,017	41.35%	57.36%	1.29%	45.53	11.10	0.99%	32.24%	20.29%	36.71%	6.25%	0.84%	2.68%
Norwegian	328	82.93%	16.77%	0.30%	40.48	13.34	7.65%	61.77%	18.35%	10.09%	0.61%	0.92%	0.61%
Portuguese (Portugal)	387	71.83%	26.87%	1.29%	33.13	14.63	24.55%	42.12%	20.93%	11.37%	1.03%	0.00%	0.00%
Portuguese (Brazil)	411	72.02%	27.49%	0.49%	37.92	13.17	13.63%	66.42%	16.06%	3.89%	0.00%	0.00%	0.00%
Russian	2,172	71.65%	27.34%	1.01%	27.42	11.57	1.34%	32.90%	41.38%	20.55%	3.23%	0.37%	0.23%

Slovak	272	88.97%	11.03%	0.00%	35.17	13.30	8.55%	51.30%	26.39%	12.27%	0.37%	0.37%	0.74%
Swedish	139	81.29%	15.11%	3.60%	42.12	14.88	8.70%	55.80%	23.19%	9.42%	2.90%	0.00%	0.00%
Turkish	146	68.49%	30.82%	0.68%	23.73	7.47	4.11%	38.36%	2.05%	55.48%	0.00%	0.00%	0.00%
Ukrainian	208	64.42%	35.10%	0.48%	32.38	10.31	12.02%	82.21%	1.44%	3.85%	0.00%	0.00%	0.48%
Chinese	232	64.22%	33.62%	2.16%	35.16	9.83	6.90%	87.93%	2.16%	3.02%	0.00%	0.00%	0.00%

Table S2

Significant unique factor loading parameters reported in the Conspiratorial Thinking Scale

	CTS Item 1	CTS Item 2	CTS Item 3	CTS Item 4
Bulgarian			**-.52**	
Czech				
German				
English				
Spanish (Colombia)				
Spanish (Costa Rica)				
Spanish (Ecuador)				
Spanish (Spain)				
Spanish (Guatemala)				
Spanish (Uruguay)				
Spanish (Honduras)				
Estonian				
Finnish				
Italian				
Japanese				
Norwegian				
Portuguese (Portugal)				
Portuguese (Brazil)				**.77**
Russian				
Slovak	**-.66**			**-.77**
Swedish				
Turkish			**-.67**	
Ukrainian				
Chinese	**-.61**			

Note. Values demonstrate the difference between the overall factor loading and group-specific factor loading in each item. Only the values from groups/items reported significant unique parameters were included in the table.

Table S3

Significant unique intercept parameters reported in the Conspiratorial Thinking Scale

	CTS Item 1	CTS Item 2	CTS Item 3	CTS Item 4
Bulgarian	**.83**	**-.50**		**.67**
Czech				
German			**-.52**	
English			**-.61**	
Spanish (Colombia)	**-.59**			
Spanish (Costa Rica)				
Spanish (Ecuador)				
Spanish (Spain)	**-.59**			
Spanish (Guatemala)				
Spanish (Uruguay)	**.85**			
Spanish (Honduras)				
Estonian	**.59**			
Finnish				**.73**
Italian				
Japanese	**.68**			
Norwegian		**.67**	**-.75**	
Portuguese (Portugal)				
Portuguese (Brazil)	**-.89**			
Russian	**.83**			
Slovak				
Swedish	**-.82**	**.58**		**-1.47**
Turkish				
Ukrainian	**.87**			
Chinese				

Note. Values demonstrate the difference between the overall intercept and group-specific intercept in each item. Only the values from groups/items reported significant unique parameters were included in the table.

Table S4

Significant unique factor loading parameters reported in the Anti-Expert Sentiment Scale

	AESS Item 1	AESS Item 2	AESS Item 3
Bulgarian			
Czech			
German			
English			
Spanish (Colombia)			
Spanish (Costa Rica)	**.77**		
Spanish (Ecuador)			
Spanish (Spain)			
Spanish (Guatemala)			
Spanish (Uruguay)			
Spanish (Honduras)			
Estonian			
Finnish			
Italian			
Japanese			
Norwegian			
Portuguese (Portugal)		**.60**	
Portuguese (Brazil)			
Russian			
Slovak			
Swedish			
Turkish			
Ukrainian	**-.79**		**-.76**
Chinese		**1.38**	

Note. Values demonstrate the difference between the overall factor loading and group-specific factor loading in each item. Only the values from groups/items reported significant unique parameters were included in the table.

Table S5

Significant unique intercept parameters reported in the Anti-Expert Sentiment Scale

	AESS Item 1	AESS Item 2	AESS Item 3
Bulgarian			
Czech			**.54**
German			
English			
Spanish (Colombia)			
Spanish (Costa Rica)		**-.88**	**-.56**
Spanish (Ecuador)			
Spanish (Spain)			
Spanish (Guatemala)		**-.26**	
Spanish (Uruguay)			
Spanish (Honduras)			
Estonian			**-.56**
Finnish		**.56**	
Italian			
Japanese			**.72**
Norwegian			
Portuguese (Portugal)	**.54**		
Portuguese (Brazil)			**-.60**
Russian			
Slovak			
Swedish			**.90**
Turkish	**.57**		**-.76**
Ukrainian	**-.51**	**1.58**	
Chinese			

Note. Values demonstrate the difference between the overall intercept and group-specific intercept in each item. Only the values from groups/items reported significant unique parameters were included in the table.

Table S6

Factor loadings estimated by multigroup CFA

	CTS Item 1	CTS Item 2	CTS Item 3	CTS Item 4	AESS Item 1	AESS Item 2	AESS Item 3
Bulgarian	1.62	1.36	1.07	1.63	1.38	1.19	1.20
Czech	1.27	1.00	1.10	1.48	1.14	.85	.90
German	.90	.97	1.26	1.16	1.01	1.00	.83
English	1.39	.96	1.39	1.70	1.14	1.46	.81
Spanish (Colombia)	1.06	1.02	1.30	1.49	1.14	.72	.66
Spanish (Costa Rica)	1.18	1.07	1.27	1.59	1.31	.45	.63
Spanish (Ecuador)	1.15	1.21	1.33	1.34	.92	.82	.73
Spanish (Spain)	.93	1.09	1.46	1.27	1.14	.98	.69
Spanish (Guatemala)	1.17	.84	.86	1.49	.90	.42	.69
Spanish (Uruguay)	1.38	1.23	1.37	1.20	.62	.73	.70
Spanish (Honduras)	1.13	.97	.97	1.29	.66	1.26	.92
Estonian	1.06	1.37	1.33	1.05	1.10	.73	.52
Finnish	.93	1.51	1.54	1.38	1.11	.92	.77
Italian	1.06	1.25	1.58	1.47	.94	1.37	1.08
Japanese	1.14	1.25	1.22	1.21	.70	1.01	.69
Norwegian	1.04	1.16	1.24	1.11	.85	.91	.87
Portuguese (Portugal)	.85	1.07	1.26	1.18	.74	1.17	.63
Portuguese (Brazil)	.73	.69	.93	1.40	.90	.70	.62
Russian	1.27	1.21	1.31	1.32	.62	1.15	1.02
Slovak	1.30	1.24	1.41	1.33	.83	.85	.92
Swedish	.56	1.51	1.46	.55	.94	.86	.97
Turkish	1.42	1.17	.77	1.64	.91	1.21	.89
Ukrainian	1.27	1.38	1.44	1.34	.40	2.28	.18
Chinese	.64	1.42	1.54	1.34	.57	1.41	.50

Table S7

Intercepts estimated by multigroup CFA

	CTS Item 1	CTS Item 2	CTS Item 3	CTS Item 4	AESS Item 1	AESS Item 2	AESS Item 3
Bulgarian	4.00	4.81	4.78	4.50	4.52	3.12	3.41
Czech	2.21	4.59	4.43	2.80	3.36	2.35	2.62
German	1.75	4.10	2.56	1.91	2.90	2.18	2.21
English	2.66	5.00	3.87	3.22	3.33	2.54	2.21
Spanish (Colombia)	2.31	5.15	4.71	3.43	3.04	2.01	1.77
Spanish (Costa Rica)	3.00	5.43	5.13	4.01	3.19	1.81	1.80
Spanish (Ecuador)	2.75	4.84	4.39	3.60	3.16	2.16	2.08
Spanish (Spain)	2.25	5.15	4.52	2.96	3.06	2.10	2.21
Spanish (Guatemala)	2.77	5.29	5.19	3.79	3.53	2.10	2.02
Spanish (Uruguay)	2.67	4.43	3.44	2.66	3.16	1.69	1.71
Spanish (Honduras)	3.64	5.39	5.12	4.59	4.01	2.64	2.56
Estonian	1.92	2.69	2.64	1.97	3.05	2.11	1.83
Finnish	1.62	3.08	2.70	2.24	2.46	2.32	1.73
Italian	1.95	4.53	3.96	2.83	3.87	2.36	2.48
Japanese	3.49	4.59	4.57	3.73	3.75	2.53	3.34
Norwegian	1.82	4.04	2.14	1.78	2.39	1.71	1.91
Portuguese (Portugal)	1.93	4.42	3.26	2.26	4.31	2.17	2.10
Portuguese (Brazil)	1.83	5.30	4.72	2.59	2.79	1.93	1.55
Russian	3.82	4.84	4.41	4.00	4.66	3.38	3.64
Slovak	2.29	4.38	4.02	2.85	3.16	2.15	2.10
Swedish	1.40	3.35	2.70	1.43	2.08	1.50	1.74
Turkish	3.41	5.29	5.10	4.07	4.67	2.95	2.07
Ukrainian	2.01	3.00	2.82	1.92	3.24	2.01	2.79
Chinese	2.17	4.04	3.84	3.13	4.28	3.34	3.11

Table S8

Factor loadings after measurement alignment

	CTS Item 1	CTS Item 2	CTS Item 3	CTS Item 4	AESS Item 1	AESS Item 2	AESS Item 3
Bulgarian	1.26	1.06	.83	1.28	.96	.82	.83
Czech	1.27	.99	1.09	1.47	1.06	.79	.83
German	.97	1.05	1.36	1.26	1.01	.99	.83
English	1.35	.93	1.35	1.65	.95	1.21	.67
Spanish (Colombia)	1.11	1.06	1.35	1.56	1.43	.90	.83
Spanish (Costa Rica)	1.26	1.13	1.35	1.69	1.72	.59	.83
Spanish (Ecuador)	1.16	1.23	1.35	1.37	1.05	.93	.83
Spanish (Spain)	.87	1.01	1.36	1.17	1.08	.93	.65
Spanish (Guatemala)	1.27	.90	.93	1.61	1.08	.51	.83
Spanish (Uruguay)	1.36	1.22	1.35	1.19	.73	.86	.83
Spanish (Honduras)	1.26	1.08	1.08	1.44	.60	1.13	.83
Estonian	1.08	1.39	1.35	1.06	1.41	.93	.67
Finnish	.82	1.33	1.35	1.21	1.20	.99	.83
Italian	.91	1.07	1.36	1.26	.72	1.05	.83
Japanese	1.27	1.39	1.35	1.35	.84	1.21	.83
Norwegian	1.13	1.26	1.35	1.21	.81	.87	.83
Portuguese (Portugal)	.92	1.15	1.35	1.27	.97	1.53	.83
Portuguese (Brazil)	1.07	1.00	1.36	2.05	1.19	.93	.83
Russian	1.31	1.25	1.35	1.36	.50	.93	.83
Slovak	1.25	1.19	1.35	1.28	.76	.77	.83
Swedish	.52	1.40	1.35	.51	.81	.74	.83
Turkish	1.27	1.04	.69	1.46	.85	1.13	.83
Ukrainian	1.20	1.30	1.35	1.26	.16	.93	.07
Chinese	.57	1.25	1.36	1.18	.94	2.31	.83

Table S9

Intercepts after measurement alignment

	CTS Item 1	CTS Item 2	CTS Item 3	CTS Item 4	AESS Item 1	AESS Item 2	AESS Item 3
Bulgarian	4.00	4.81	4.78	4.50	4.52	3.12	3.41
Czech	3.09	5.28	5.19	3.82	4.46	3.17	3.49
German	3.11	5.57	4.46	3.67	3.85	3.11	2.99
English	3.16	5.35	4.37	3.83	3.87	3.23	2.60
Spanish (Colombia)	2.57	5.40	5.03	3.79	4.44	2.89	2.58
Spanish (Costa Rica)	2.87	5.31	4.99	3.83	4.41	2.24	2.39
Spanish (Ecuador)	3.20	5.31	4.91	4.12	4.11	3.00	2.83
Spanish (Spain)	2.58	5.53	5.02	3.40	4.31	3.17	2.96
Spanish (Guatemala)	2.79	5.31	5.20	3.81	4.57	2.59	2.82
Spanish (Uruguay)	4.01	5.63	4.77	3.83	4.18	2.90	2.87
Spanish (Honduras)	3.27	5.08	4.81	4.18	4.29	3.17	2.94
Estonian	3.75	5.06	4.95	3.79	4.23	2.90	2.39
Finnish	3.20	5.62	5.30	4.56	4.11	3.68	2.87
Italian	2.77	5.50	5.18	3.97	4.55	3.34	3.26
Japanese	3.84	4.97	4.94	4.10	4.09	3.02	3.67
Norwegian	3.56	5.98	4.23	3.65	3.77	3.18	3.32
Portuguese (Portugal)	2.97	5.72	4.79	3.70	4.77	2.89	2.49
Portuguese (Brazil)	2.27	5.71	5.27	3.43	3.94	2.82	2.35
Russian	4.00	5.01	4.59	4.18	4.49	3.05	3.35
Slovak	3.27	5.31	5.08	3.85	4.20	3.21	3.24
Swedish	2.34	5.89	5.17	2.36	4.13	3.38	3.85
Turkish	3.16	5.08	4.97	3.78	4.80	3.12	2.19
Ukrainian	4.03	5.19	5.11	4.05	3.71	4.70	3.00
Chinese	2.71	5.23	5.14	4.26	4.23	3.20	3.06

Table S10

Correlation between conspiratorial thinking and anti-expert sentiment with trust (before measurement alignment)

	1	2	3	4	5	6	7	8
1. Conspiratorial Thinking								
2. Anti-expert Sentiment	.44							
3. Trust in parliament/government	-.42	-.17						
4. Trust in police	-.38	-.19	.70					
5. Trust in civil service	-.41	-.22	.74	.75				
6. Trust in health system	-.39	-.29	.56	.66	.69			
7. Trust in the WHO	-.39	-.34	.42	.37	.47	.49		
8. Trust in governmental effort	-.39	-.19	.79	.61	.68	.56	.46	
9. Trust in scientific research community	-.43	-.44	.39	.39	.46	.54	.62	.46
